# Probing the Nanoscale Onset of Plasticity in Electroplated Copper for Hybrid Bonding Structures via Multimodal Atomic Force Microscopy

**DOI:** 10.1021/acsanm.5c05142

**Published:** 2025-12-09

**Authors:** Nicolas A. Alderete, Paresh D. Daharwal, Cristian V. Ciobanu, Gheorghe Stan

**Affiliations:** † Material Measurement Laboratory, 10833 National Institute of Standards and Technology, Gaithersburg, Maryland 20899, United States; ‡ School of Science and Engineering, The George Washington University, Washington, D.C. 20052, United States; § Advanced Packaging Technology and Manufacturing, 7198 Intel Corporation, Hillsboro, Oregon 97124, United States; ∥ Department of Mechanical Engineering and Materials Science Program, 3557 Colorado School of Mines, Golden, Colorado 80401, United States

**Keywords:** atomic force microscopy, nanoscale plasticity, copper, advanced packaging, hybrid bonding

## Abstract

The slowdown of Moore’s law has elicited a paradigm shift whereby shrinking of in-plane dimensions is being replaced by 3D-stacking advanced packaging approaches to satisfy the ever-increasing demands for power, performance, area, and cost. Driven by the widespread use of metallic interconnects with submicron pitches, robust metrology for probing mechanical behavior at the nanoscale emerges as a key area of interest in the semiconductor industry. Here, we develop an atomic force microscopy (AFM)-based protocol for characterizing the incipient stages of plasticity and illustrate it on hybrid bonding-ready (prior to bonding) copper pads. We combined AFM’s high-resolution imaging with contact resonance and indentation techniques (including single- and multistep indentation) to characterize the mechanical heterogeneity of the material, quantify the nanoscopic yield stress statistics, and derive indentation stress–strain curves. From these measurements, we have clarified the mechanisms of early plasticity and determined the elastoplastic constitutive response of polycrystalline copper, including parameters such as elastic modulus, yield stress, and strain-hardening slope. Besides providing metrology relevant to various length scales, our approach offers a pathway to utilize an industry-standard instrument for characterizing the thermomechanical properties that are essential for the development of semiconductor structures.

## Graphical abstract

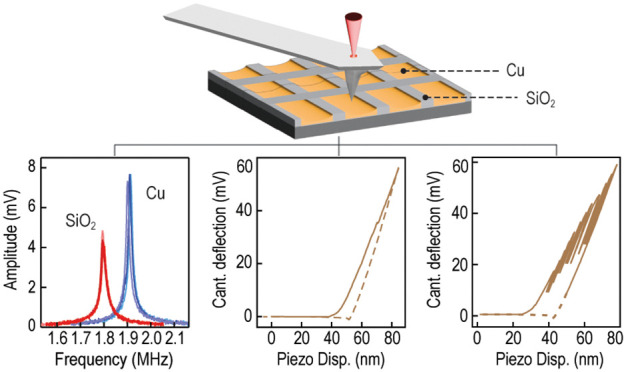

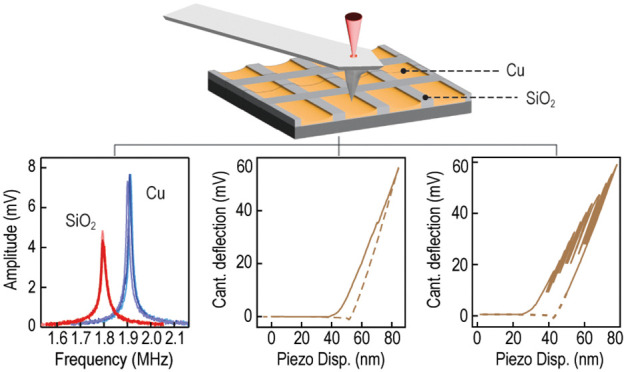

## 1 Introduction

The advancement of semiconductor technology cannot continue solely under the paradigms of Moore’s and More Moore’s laws, as dimensions approach the physical limits of transistor scaling. Nonetheless, ongoing developments in fields such as artificial intelligence, large-scale computing, big data, and robotics are driving the need for improvements in power, performance, area, and cost (PPAC).^1^ In this context, various advanced packaging techniques (an umbrella term for different architectural accelerators) have emerged as potential solutions to sustain miniaturization trends and meet PPAC requirements^2^ (Figure 1a). One promising approach is hybrid bonding technology, which enables direct metal–metal and dielectric-dielectric connections with interconnect pitches <10 μm, without the need for wires or bumps in the circuit.^3^ In a general hybrid bonding process (Figure 1b), two identical dies made of silicon (Si) and silicon dioxide (SiO_2_) are prepared with embedded copper (Cu) pads.^4^ During a chemical-mechanical polishing (CMP) step, the dielectric is smoothened and the copper on each die is recessed slightly (i.e., dishing) to ensure it does not impede the dielectric–dielectric bonding. Heat and pressure are subsequently applied, causing the Cu pads to expand, fill the recesses, and contact the opposing Cu pads. These contacts lead to copper migration and bonding of the two dies. Thus, two of the most critical steps in hybrid bonding, CMP and annealing plus bonding phase, involve nanoscale mechanical and thermomechanical processes. During CMP, the copper is deformed and abraded by contact with silica particles in a slurry.^5,6^ In the annealing and bonding phase, the asperities at the mating interfaces come into contact and plastically deform, increasing the effective merging area for an enhanced diffusion-assisted bonding.^7^ Therefore, understanding and improving these processes requires a thorough characterization of the elastic and plastic properties of bonding-ready copper at the nanoscale,^1,8,9^ which differ from those measured at the macro-, meso- and microscale due to the emergence of size^10^ and processing effects.^11,12^

**Figure 1:**
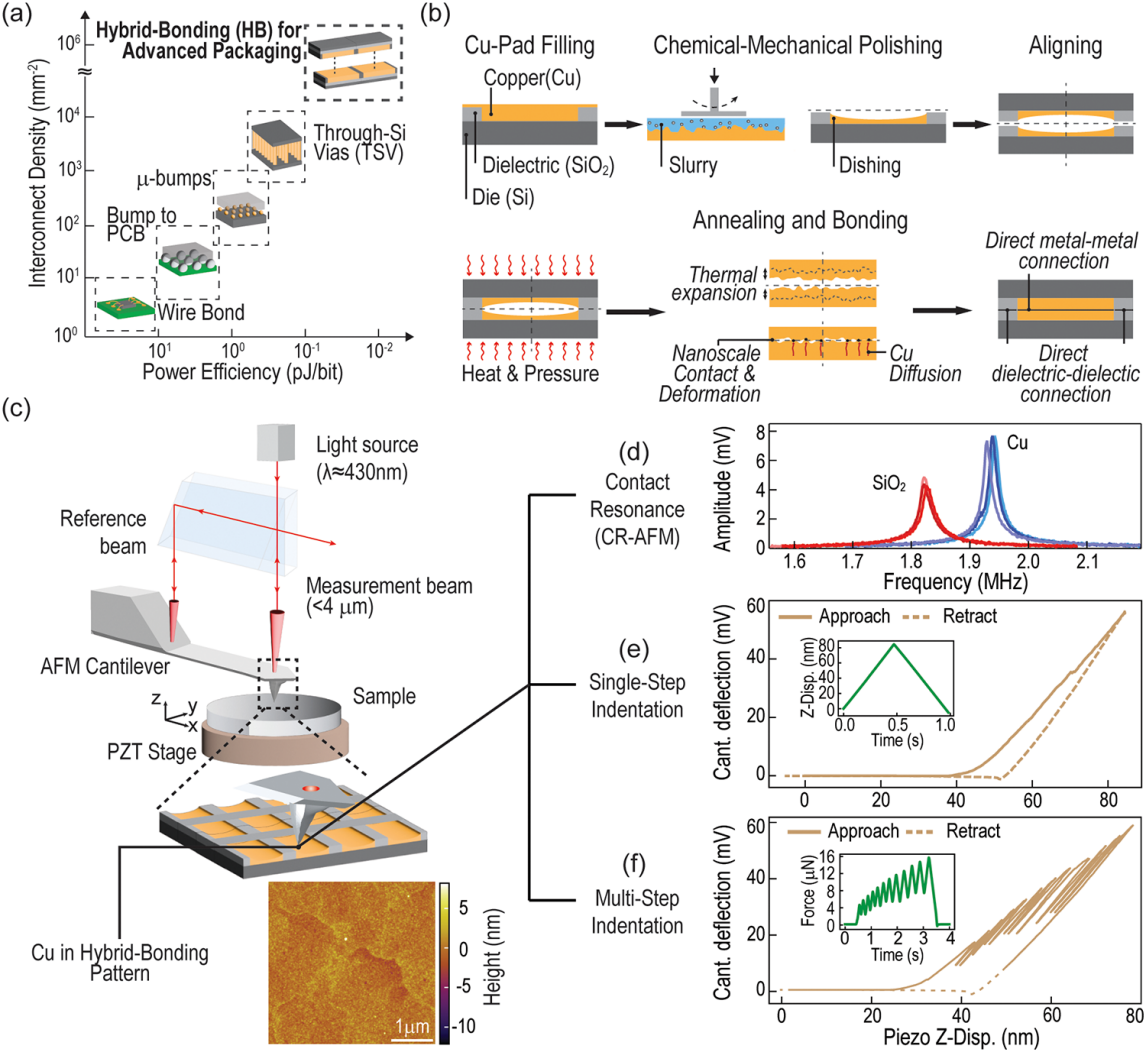
(a) Overview of scaling trends in 3D-interconnect technologies in terms of energy efficiency and interconnect density; (b) schematic representation of the main steps involved in a typical hybrid bonding process. Multimodal AFM experiments on hybrid-bonding ready patterns: (c) experimental setup for interferometric AFM, along with a topographic image of copper in a hybrid bonding pattern (prior to bonding). Representative experimental curves obtained through (d) CR-AFM, (e) single-step, and (f) multistep AFM indentation. Insets in (e) and (f) show the imposed displacement/load profiles as a function of time.

Fundamental micro- and nanoscale plasticity studies in copper have primarily been conducted using conventional nanoindentation,^13–16^ atomic force microscopy (AFM) indentation,^17^ miniaturized uniaxial testing,^18,19^ and forward and inverse computational methods.^13,20–22^

Despite the versatility and advanced capabilities of modern AFMs for nanoscale mechanical mapping,^23^ there is a significant lack of studies leveraging AFM for the characterization of nanoscale incipient plasticity in materials specific to the semiconductor industry. Most studies using AFM for mechanical characterization in heterogeneous integration and advanced packaging have primarily focused on topographic imaging to infer the mechanical response of the samples,^24–26^ rather than directly probing their nanomechanical properties. For example, observation of thermoplastic deformation in hybrid-bonding-ready Cu-SiO_2_ structures can be simply observed by AFM topographical scans as a function of temperature.^24^ However, to understand and accurately model the thermomechanical response of these structures, we require direct measurements of their material parameters at the nanoscale, rather than relying on values obtained from macroscale tests. These needs align with the continued downward trend in size-scaling and the critical need for application-relevant robust metrology as identified in the International Roadmap for Devices and Systems.^1^

We address these gaps by introducing a multimodal AFM-based metrology framework designed to characterize the nanoscale elastic and plastic response of copper at room temperature, specifically within actual hybrid bonding patterns prior to the bonding process (Figure 1b). In this framework, we utilize contact resonance (CR-AFM, Figure 1d) and AFM-indentation (Figure 1e,f) together with the topographic capabilities of AFM to measure local moduli, determine yield stresses, and derive indentation stress–strain curves at room temperature. Our multimodal AFM approach, with tip radii less than 40 nm, enables probing the mechanics of nanoscale volumes on precise nanostructural surface features such as grain boundaries, without requiring additional sample preparation. A key advancement of this work is the ability to resolve subnm size pop-in events taken at force increments <1 μN. Furthermore, our AFM-based strategy lays the groundwork for future nanoscale hybrid-metrology that combines topographic, mechanical, electrical, and thermal capabilities.^27^ These types of measurements and corresponding data pertaining to structures prepared for integration are essential for enhancing predictive manufacturing in hybrid bonding applications.

## 2 Experimental Section

### 2.1 Hybrid Bonding Samples

Samples were collected from wafers featuring hybrid bonding SiO_2_/Cu patterns (Intel Corporation, Hillsboro, OR, USA) that underwent chemical-mechanical polishing. The surface of the polycrystalline Cu pads exhibits micrometer-sized grains. In AFM topographic images, these grain boundaries are visible to varying extents, with height differences across them measuring a few nanometers.

### 2.2 AFM Topographic Imaging

AFM experiments were performed using an environmental Vero AFM (Oxford Instruments/Asylum Research, Santa Barbara, CA, USA). The Vero AFM features a built-in Quadrature Phase Differential Interferometer (QPDI),^55^ which directly measures the vertical displacement of the tip based on the wavelength λ of the light (Figure 1c). In addition to benefiting from low-noise measurements through interferometric detection, the QPDI provides two significant advancements: (1) the vertical deflection measurement directly reflects the AFM tip displacement, improving the characterization of the tip–sample interaction, and (2) the cantilever deflection is converted into displacement without requiring contact with the sample, enabling stiffness calibration through the thermal tune method prior to contact. Furthermore, unlike the optical beam deflection method commonly used in AFM, QPDI minimizes crosstalk between lateral and vertical forces. All experiments were conducted in air at room temperature. The uncertainties reported correspond to the experimental standard deviation of the measurements taken during each experiment (see Supporting Information, Notes S1 and S4–S6).

Topographic imaging of selected areas within the hybrid bonding patterns was conducted in noncontact mode, both before and after AFM indentations, using the same tip for both operations. For high-resolution imaging following the indentations, sharper AFM tips were utilized. In these instances, PPP-SEIH silicon cantilever tips (Nanosensors, Neuchâtel, Switzerland) were used, with cantilever stiffness of around 8.5 N/m and tip radius less than 10 nm. All topographic images were processed using first-degree polynomial flattening.

### 2.3 Contact Resonance AFM

CR-AFM measurements were conducted using photothermal excitation of the cantilever in dual AC mode. A DT-NCHR probe (Nanosensors, Neuchâtel, Switzerland), with a resonance frequency in air *f*_1_^air^ = 415.8 kHz and a spring constant *k*_c_ = 9.2 N/m, was used. Frequency maps were generated over a Cu pad surrounded by SiO_2_, with an applied force of 750 nN, a frequency scan rate of 0.5 Hz, and a resolution of 512 px × 512 px. During the mapping, the two driving frequencies were kept apart at a constant difference of 120 kHz. A complete description of the experimental and analysis methodology is presented in Supporting Information, Note S1.

### 2.4 AFM-Indentation

Single-step and multistep AFM-indentation measurements were performed using silicon probes with conospherical diamond tips (Adama Innovations Ltd., Dublin, Ireland), and spring constants *k*_c_ ranging between 508 and 528 N/m. The shape of the tips was regularly assessed against a TGT1 test grating (K-TEK Nanotechnology, Wilsonville, OR, USA) in between indentation arrays to check for significant wear or damage. Throughout the measurements, the tips maintained a spherical end, with only a slight increase in tip radius observed after hundreds of measurements (see Supporting Information, Note S7). Force versus indentation depth, *P* vs *h*, curves were derived from the cantilever deflection voltage *V* versus piezo displacement *d* curves, *V* vs *d*, with *P* = *k*_c_ *h* and *h* = *d* – *VS*, *S* being the deflection sensitivity of the cantilever in nm/V. The processing of the force-spectroscopy data was carried out using an in-house developed script in Python, which utilized the SciPy^56^ and Scikit-learn^57^ packages.

#### 2.4.1 Single-Step AFM-Indentation

Single-step indentations were performed as square arrays over areas of approximately 4 μm × 4 μm, at a given maximum applied load. The loading and unloading segments of the indentations were carried out at a velocity of 200 nm/s, with a sampling rate of 500 Hz, and a scan rate of 1 Hz. Force–displacement curves were analyzed for several metrics like pop-ins, total work, plastic work, residual depth, and cumulative excursion length. Pop-ins (i.e., small displacement excursions on the loading curves) were identified with the aid of an in-house developed algorithm. A subsequent visual inspection was conducted to detect any potentially overlooked pop-ins and to ensure accuracy. The force–displacement coordinates of all identified pop-ins, as well as the total number of pop-ins, were recorded for statistical analysis. A complete description of the experimental methodology and analysis is presented in Supporting Information, Note S2.

#### 2.4.2 Multi-Step AFM-Indentation

Multistep AFM-indentation experiments were performed over selected areas within Cu pads. Typically, the maximum load used for these tests was around 15 μN, with approximately 20 partial loading–unloading cycles made to gradually reach the desired maximum load. Partial unloading was performed up to 25%of the cycle’s maximum load to maintain a conformal tip–sample contact during unloading. The loading and unloading segments were executed at velocities of 50 nN/s, and with a sampling rate of 500 Hz. Indentation stress–strain curves were derived following the formalism developed by Pathak and Kalidindi,^49^ based on the analysis of the unloading segments. A complete description of the experimental methods and analysis is presented in Supporting Information, Note S3.

## 3 Results and Discussion

### 3.1 Elastic Modulus Characterization

The elastic modulus of polycrystalline Cu pads was determined using contact resonance atomic force microscopy (CR-AFM) mapping, with results presented in Figure 2. In essence, CR-AFM utilizes the resonant frequency shift of the AFM cantilever while in elastic contact with the sample to quantify the elastic and viscoelastic properties of materials at the nanoscale.^23,28^ We performed CR-AFM measurements on both Cu pads and adjacent SiO_2_ regions, over areas of about 25 μm^2^ on each material. The measurements taken in the SiO_2_ regions served as a reference for converting the measured CR frequencies into indentation moduli^28,29^ (Figure 2a,b). The indentation modulus *M* is defined as *M* = *E*/(1 – ν^2^), where *E* and ν represent the Young’s modulus and Poisson’s ratio, respectively. For the reference SiO_2_ material, a Young’s modulus *E*_SiO2_ = 72 GPa and a Poisson’s ratio ν_SiO2_ = 0.17 were assumed, which correspond to an indentation modulus of *M*_SiO2_ = 74.1 GPa. The indentation modulus of the tip (diamond) was taken as *M*_Tip_ = 1250 GPa, obtained from *E*_Tip_ = 1200 GPa and ν_Tip_ = 0.2. Using these values, we calculated the reduced elastic modulus of the SiO_2_ as *E*_eff, SiO2_ = (1/*M*_SiO2_ + 1/*M*_Tip_)^−1^.

**Figure 2:**
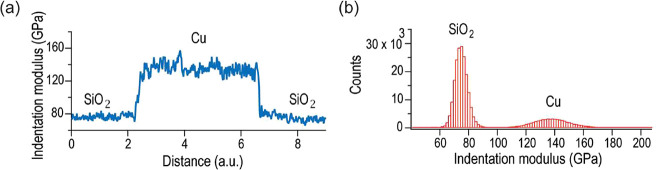
CR-AFM measurements. (a) Single-line profile of indentation moduli across SiO_2_ and Cu regions as determined by CR-AFM mapping, (b) histogram of the indentation moduli obtained from CR-AFM over the mapped SiO_2_ and Cu regions.

The modulus profile shown in Figure 2a exhibits minimal variation across the reference SiO_2_ regions, with an indentation modulus of *M*_SiO2_^Exp^ = 74.1 GPa ± 4.1 GPa. The uncertainty in the measurements of SiO_2_ is ascribed to the inherent variability of the contact area during CR-AFM mapping. In contrast, a more pronounced variation in indentation modulus is observed on the profile of the Cu area (*M*_Cu_^Exp^ = 136.4 GPa ± 11.5 GPa), as also evidenced in the histogram of Figure 2b. This distinct contrast within the Cu pad can be attributed to its polycrystalline structure. The range of indentation moduli shown in Figure 2b can be correlated with the indentation moduli of the three main crystallographic directions of Cu, whose theoretical indentation moduli are 129, 138, and 141 GPa, for <100>, < 110>, and <111>, respectively.^30^ Although a predominance of the <111> texture is generally expected due to its greater diffusivity (as Cu–Cu hybrid bonding is a diffusion-based process), the observed heterogeneity is not surprising because the process parameters associated with various fabrication steps of the Cu pads (such as Cu-seeding via physical vapor deposition, Cu-electroplating, annealing, and CMP) are known to influence their texture.^31–33^ Establishing a clear association between the mechanical properties and specific crystallographic orientations is less important for the purposes of this work. Instead, we focus on using these results to develop an elastic-plastic characterization that is highly localized and can be effectively performed at specific locations on samples of interest.

### 3.2 Incipient Plasticity Characterization

With the indentation moduli determined from CR-AFM, we now turn our attention to the onset of plastic deformation, also known as incipient plasticity. As briefly noted in the introduction, AFM-based force-spectroscopy is particularly well-suited for studying the onset of plasticity due to its ability to probe material volumes on the nanometer scale, where the likelihood of encountering and moving dislocations is relatively low.^34^ In turn, AFM-indentation allows for the investigation of dislocation nucleation mechanisms^10^ and defect-induced plastic mechanisms.

Single-step indentations were first performed on the center region of two separate Cu pads from the same batch of fabrication. Figure 3a shows a topographic scan of the surface of one of the pads after indentation, where the pad’s polycrystalline structure and some surface defects can be observed. Individual grains in the AFM topography can be distinguished from one another due to the slight misalignment of their polished surfaces. The significant nonuniformities observed across polycrystalline copper grains arise from the varying mechanical wear rates during CMP, which depend on the crystallographic orientation of the grains.^35,36^ Additionally, small, thin ridges (measuring less than 1 nm) can be observed along the grain boundaries, resulting from the mechanical mismatch between the grains. As shown in Figure 3a, the postimaging AFM offers high spatial resolution for locating indents with respect to the interior of the grains and their boundaries. Force-depth curves, one of which is presented in Figure 3a, display similar characteristics, featuring a jagged loading segment and a relatively smooth unloading curve. The former is associated with the occurrence of pop-in events related to plastic deformations (indicated by black arrows in Figure 3a), while the latter is indicative of elastic unloading without additional effects, such as pop-outs.

**Figure 3:**
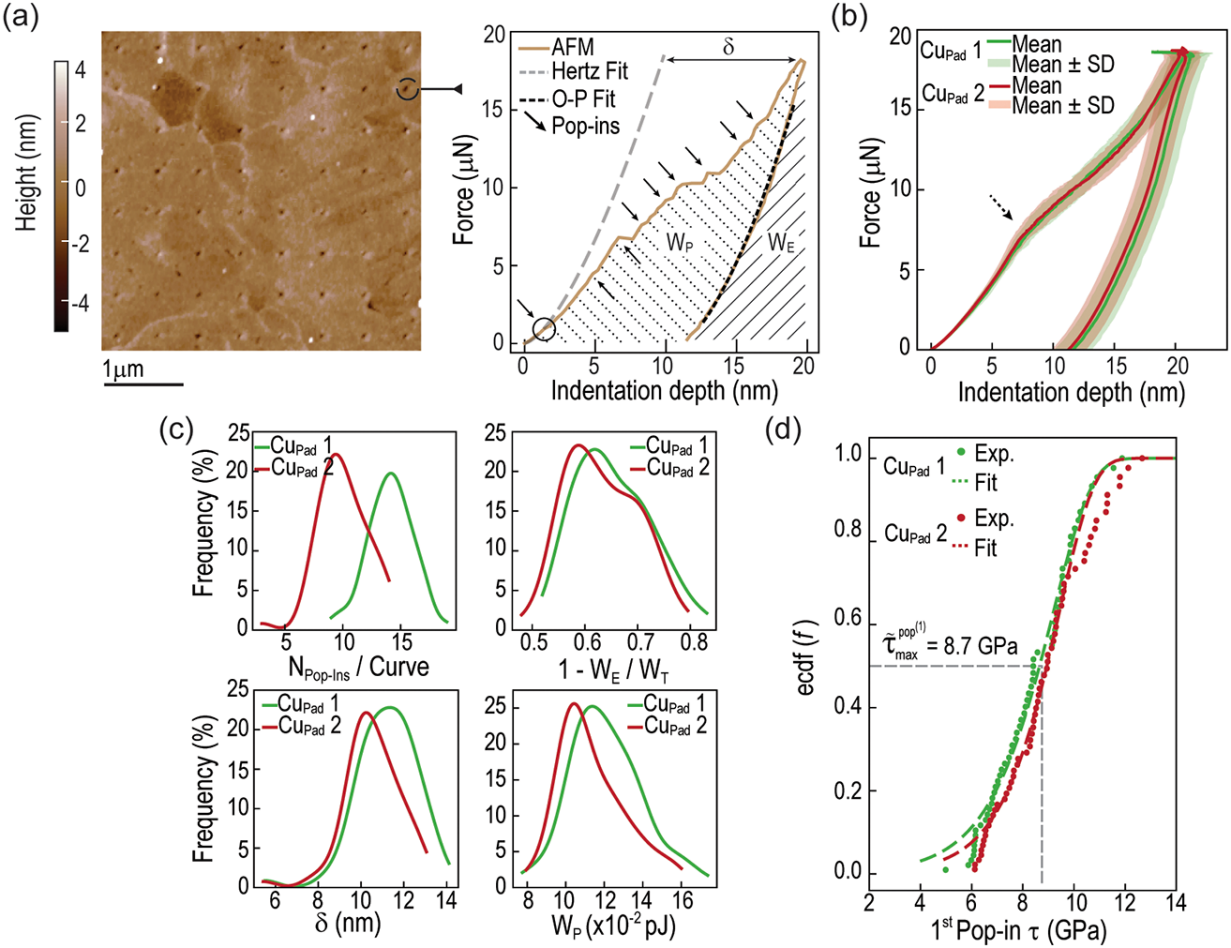
AFM single-step indentation characterization on Cu pads. (a) Postindentation topographic scan with a selected force–displacement curve shown. (b) Average force–indentation depth curves, with the shaded region covering curves within one standard deviation. (c) Histogram fits for plastic metrics including the number of pop-ins per indentation curve, plasticity index, cumulative excursion length, and plastic work. (d) Empirical cumulative distribution (ecdf) of maximum shear stress at the first pop-in event, as defined by eq 2.

Averaging the force-depth curves obtained from each pad (Figure 3b) provides a clearer understanding of their behavior under load and shows that our AFM indentations are consistent across pads. In the average curves, the pop-ins are smoothed out, and an inflection is observed at approximately 7.5 μN force (black dashed arrow in Figure 3b). Before reaching this point, the (average) loading curve resembles the unloading path. After the inflection at 7.5 μN, the material exhibits a significantly more compliant response. By comparing the average curves with the individual ones, we find that the inflection region corresponds to the occurrence of the largest pop-in events recorded during the experiments. Given that these are not the first pop-ins, the largest pop-ins likely reflect a collective motion of dislocations^37^ resulting from the interaction between pre-existing dislocations and those generated around the AFM tip.^14^ The fluctuations observed at the maximum load of the averaged profile in Figure 3b result from the sparse averaging of force–distance curves that reach the same maximum load at various indentation depths. Additional examples of individual force–distance curves are provided in the Supporting Information. Other plasticity metrics (Figure 3c) derived from individual force-depth curves also show good consistency across pads. The number of pop-in events per curve generally ranges from 8 to 15, with an average plasticity index and plastic work around 0.6 and 0.012 pJ, respectively.

As shown in the individual indentation curve (Figure 3a) and the curves in Supporting Information, Note 4, the actual departure from elasticity occurs considerably earlier in the loading path than the aforementioned inflection around 7.5 μN. This departure can be assessed by fitting the elastic segment up to the first pop-in with the classical Hertz expression for the contact of a paraboloid onto a flat, elastic half-space:^38^

$$P=\frac{4}{3}E_{\mathrm{eff}}\sqrt{R}h^{3/2}$$ 1

where *P*, *E*_eff_, *R*, *h* are the load, the effective modulus, the tip radius, and the displacement, respectively. The elastic modulus values obtained from CR-AFM measurements were used to fit eq 1, leading to an estimate of the tip radius *R* = 12.3 nm ± 1.4 nm (see Supporting Information, Notes 2 and 7). In our analysis, we disregarded any existing native oxide layer on the surface of Cu due to the lack of a distinct compliant region at the contact, which suggests that this layer may be very thin, possibly within the nanometer range. After fitting, the maximum shear stress (τ_max_^pop^(1)^^) at the point of departure from the Hertzian response (marked with a circle in Figure 3a), can be calculated as:^38^

$$\tau_{\max}^{{\mathrm{pop}}^{\left(1\right)}}=0.31\sqrt[{3}]{\frac{6E_{\mathrm{eff}}^{2}P^{{\mathrm{pop}}^{\left(1\right)}}}{\pi^{3}R^{2}}}$$ 2

where *P* ^pop^(1)^^ is the load at the first pop-in. The maximum shear stress in eq 2 represents the onset of plasticity (i.e., incipient plasticity) and can be regarded as the nanoscopic yield stress. Furthermore, by analyzing various force-depth curves (refer to Figures S4 and S5 from Supporting Information), it is evident that pop-ins occur at different load-depth pairs, highlighting their stochastic nature. In this context, Figure 3d presents the empirical cumulative distribution function of the maximum shear stresses calculated at the first pop-in events from each indentation experiment. For both Cu pads examined, the distributions are narrow, ranging from 5 to 12 GPa, with median maximum shear stress (τ̃_max_^pop^(1)^^, tilde denoting median) of 8.4 and 8.9 GPa, respectively. These values are of the order of the theoretical shear strength of copper, generally estimated to be about *G*/2π (with *G* the shear modulus of the material, which for Cu<111> is about 48 GPa).^39^ Previous experimental studies by Suresh et al.^15^ and Gouldstone et al.^40^ reported shear strengths of 20.8 and 15 GPa, respectively, through nanoindentation of primarily (111)-copper thin films on Si substrates. More recent computational^41^ and experimental^14^ works found lower shear strengths around 4.56 and 4.20 GPa under spherical nanoindentation, respectively. As such, our values fall within these upper and lower bounds, reflecting both our methodology and the characteristics of copper in hybrid bonding patterns.

To gain mechanistic insight into the nature of the first pop-in, the empirical cumulative distributions (*f*) are fitted using^42^

$$f=1-\exp\left[-\frac{\eta k_{B}T}{\mathrm{\tau ̇}v^{*}}\exp\left(\frac{\tau v^{*}}{k_{B}T}\right)\right]$$ 3

where *k*_B_, *T*, v*, η, τ, τ̇ are Boltzmann’s constant (1.380649 × 10^–23^ J/K), temperature, activation volume, defect nucleation rate, and the shear stress and rate (where τ_max_^pop^(1)^^ has been denoted τ, for brevity), respectively. The fit is performed after using eq 3 to write ln­[ln­(1/(1 – *f*)] as a linear function of τ, to obtain v* and η from the slope and intercept, respectively. From the fits (dashed lines in Figure 3d), an average value of 3.5 × 10^–3^ nm^3^ for the activation volume is obtained, on the order of 20% the atomic volume of copper (1.182 × 10^–2^ nm^3^), and consistent with the values reported for other face-centered cubic (FCC) metals.^43,44^ This correlation suggests that the plastic events, which appear as the first pop-in in the force-depth curves, are associated with the homogeneous nucleation of dislocations caused by the reconfiguration of a small number of atomic bonds, rather than a larger-scale reconfiguration of the lattice.^10,45^ Although this topic is beyond the scope of this work, future studies employing computational modeling (such as molecular dynamics) and experimental techniques (like transmission electron microscopy) could provide deeper insights into the dislocation nucleation phenomena. Additionally, the similarity between the cumulative excursion lengths (δ, Figure 3c) and the sum of abrupt displacements in the force-depth curves indicates that the plastic dissipation is primarily driven by shear.

### 3.3 Influence of Grain Boundaries

As discussed in the previous sections, Cu pads in hybrid bonding patterns are polycrystalline, raising the question of how grain boundaries influence the onset of plasticity in these samples. It is well established that grain boundaries play a significant role in accommodating deformation, controlling strength, and regulating diffusion, with mechanisms often differing from those within the grains.^46,47^ However, the reduced size of grains and their boundaries make it challenging to examine their local mechanics. In this context, the topographic capabilities of AFM and the ability to use sharp tips for force-spectroscopy enable the precise positioning of the indenter to probe grain boundary properties and compare them to those of the grains themselves. To achieve this, the plastic behavior of copper grain boundaries in hybrid bonding patterns was studied using a methodology similar to that described in Section 3.2. On a different copper pad, a set of indentations was performed on both grain boundaries and within grains, as shown in the topographic scan in Figure 4a.

**Figure 4:**
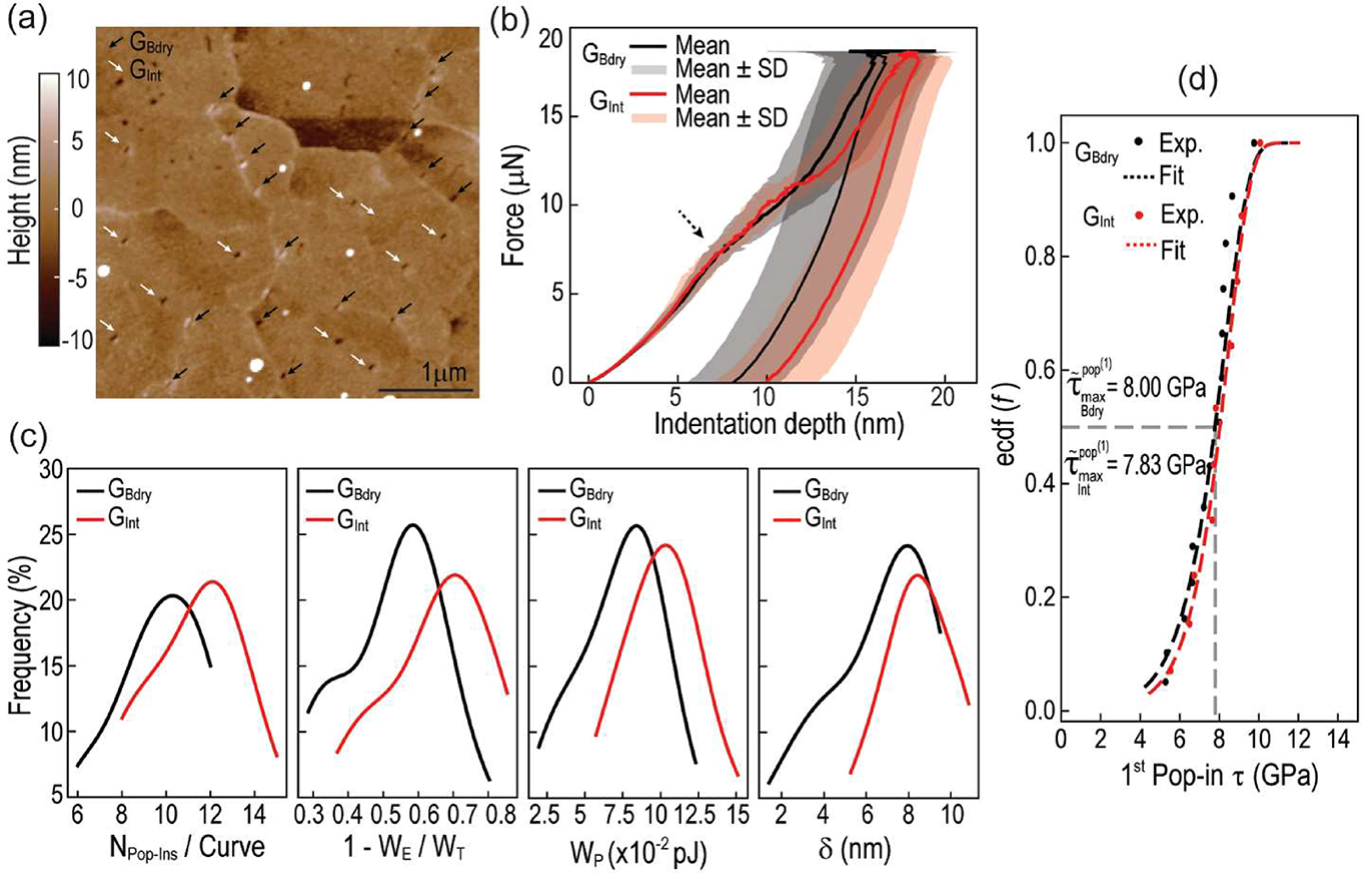
AFM single-step indentation comparison between grain boundary (*G*_Bdry_) and grain interior (*G*_Int_) plasticity. (a) Postindentation topographic scan with indents on grain boundaries and grain interiors highlighted. (b) Average force–indentation depth curves (shaded region signifies one standard deviation). (c) Histogram fits for plastic metrics: number of pop-ins per indentation curve, plasticity index, cumulative excursion length, and plastic work. (d) Empirical cumulative distribution (ecdf) of maximum shear stress at fist pop-in, as defined by eq 2.

The average force indentation-depth curves (Figure 4b) show comparable loading behavior up to nearly 7.5 μN (as indicated by the black dashed arrow in Figure 4b), consistent with the onset of plasticity discussed in Section 3.2. Additionally, the unloading paths appear parallel yet shifted, illustrating similarity in elastic behavior. The difference arises in the behavior following the kink in the average loading curve, wherein the grain boundaries exhibit a stiffer response and reduced ductility compared to the grain interiors. The latter is clearly seen when comparing the plasticity index, plastic work and cumulative departure length metrics, as shown in Figure 4c. In all cases, these metrics indicate increased plasticity for points within the grains compared to boundaries, which is consistent with the notion that grain boundaries act as barriers to dislocation motion.^48^

Notably, in the early stages of plasticity, the differences between boundary and interior points are not as pronounced, with the median maximum shear stress differing by less than 3% (8.00 and 7.83 GPa, respectively), resulting in distributions that nearly overlap around the theoretical shear strength (Figure 4d). Therefore, the influence of grain boundaries appears to be more prevalent at the post-nucleation level rather than at the stage of initial dislocation nucleation.

### 3.4 Stress–Strain Curves

We utilized our AFM multistep indentations to obtain the constitutive relations for copper in the form of indentation stress–strain (ISS) curves. To achieve this, we followed the methodology developed by Pathak and Kalidindi^49^ and fitted the unloading segments (i.e., elastic reloading segments) of multistep indentations using a more general Hertz expression:

$$P^{\left(i\right)}=\frac{4}{3}E_{\mathrm{eff}}\sqrt{R_{\mathrm{eff}}^{\left(i\right)}}{h^{\left(i\right)}}^{3/2}$$ 4

where (i) represents a given unloading segment. It is important to note that eq 4 differs from eq 1 in the effective radius term *R*_eff_^(*i*)^. This is because, in multistep experiments, an effective tip radius must be used to account for the plastic deformation of the sample. To fit eq 4 with *R*_eff_^(*i*)^ as the sole fitting parameter, it was assumed that the elastic moduli remained constant. The value of the effective radius was used to compute the contact radius as *r*_c_^(*i*)^ = (3*P*_t_^(*i*)^ *R*_eff_^(*i*)^/4*E*_eff_)^1/3^, with (*P*_t_^(*i*)^, *h*_t_^(*i*)^) the maximum load and depth at the maximum load of each segment.^50^ From this, pairs of stress–strain values were ultimately obtained as:^49^

$$\left(\varepsilon_{\mathrm{ISS}}^{\left(i\right)};\sigma_{\mathrm{ISS}}^{\left(i\right)}\right)=\left(\frac{4h_{t}^{\left(i\right)}}{3\pi r_{c}^{\left(i\right)}};\frac{P_{t}^{\left(i\right)}}{\pi {r_{c}^{\left(i\right)}}^{2}}\right)$$ 5

Figure 5a presents topographic images of the surface of Cu pads before and after multistep indentations. These indentations were performed within grains (i.e., not on grain boundaries) and the residual indentations with depths reaching nearly 20 nm. It is important to note that the postindentation residuals of multistep indentations in Figure 5a appear somewhat elongated. This effect is likely due to the material’s crystallographic anisotropy and the uneven distribution of the pile-up around the rim. More pile-up material was observed around the multistep indents compared to the single-step ones. Associated with the indents shown in Figure 5a, Figure 5b depicts two individual multistep force–indentation depth curves, with the Hertzian fits superimposed on the unloading segments (more curves presented in Supporting Information, Note 6). The force-depth data is well captured by the expression in eq 4, which facilitates the conversion to indentation stress–strain values. This is justified by virtue of the nonoverlapping loading–unloading segments, which indicate plastic deformation. In contrast, the unloading and reloading segments, on the other hand, appear overlapped, indicating negligible hysteresis.

**Figure 5:**
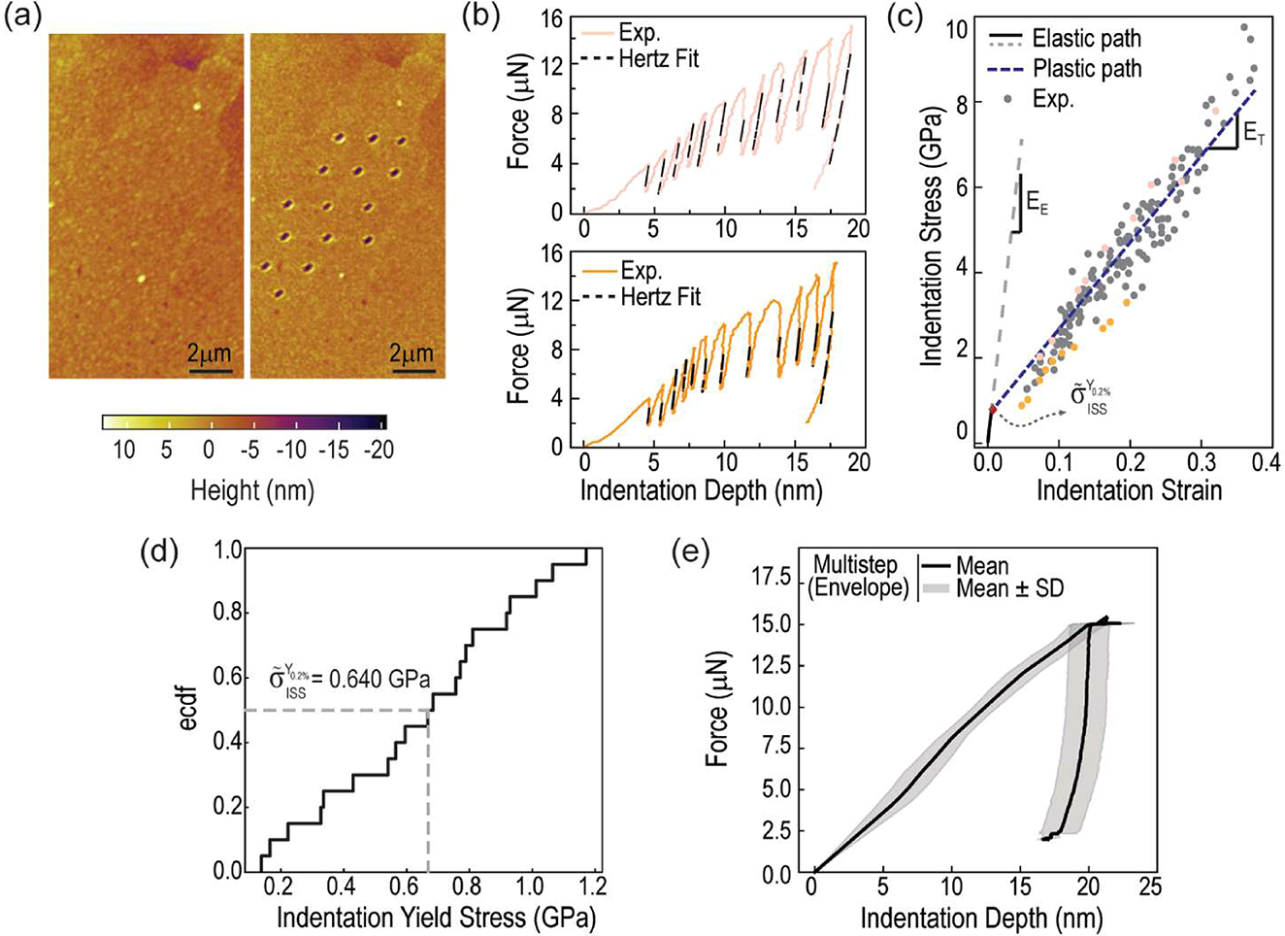
Multistep AFM-indentation experiments. (a) Before and after multistep indentation topographic scans within a Cu pad. (b) A subset of force–indentation depth curves with the Hertzian fits superimposed on the unloading segments. (c) Indentation stress–strain curve with stress–strain pairs from all experiments (gray markers) and the ones derived from the force-depth curves from (b) (orange and pink markers). (d) Empirical cumulative distribution function of the indentation yield stress from all of the experiments, with the median indentation yield stress (σ̃_ISS_^Y_0.2%_^) highlighted. (e) Average force-depth envelope curve for multistep indentation experiments (shaded region signifies one standard deviation).

By analyzing the force–depth curves from all multistep indentation experiments, a collection of indentation stress–strain pairs was obtained, as shown in the combined plot of Figure 5c. Overall, the stress–strain data aligns well with a linear trend of slope *E*_T_, which is smaller than the elastic slope *E*_E_. This indicates that the response is not solely elastic. This is further evidenced by the force–indentation depth curves, where no overlap between loading and unloading was observed during the cycles. Consequently, the slope *E*_T_ represents the linear strain-hardening behavior of the Cu pads, also referred to as tangent stiffness. We calculated the median *E*_T_ from each indentation stress–strain curve (Figure 5c) to obtain an average *E*_T_ = 20.3 GPa. From this value, by computing the intersection of the plastic path (dashed blue line in Figure 5b) with the 0.2% offset elastic path, a median indentation yield stress of σ̃_ISS_^Y_0.2%_^ = 0.64 GPa was determined. Thus, by deriving the yield stress and tangent stiffness through multistep indentations, we can elucidate the elastic-plastic constitutive behavior of the Cu pads.

The yield stress obtained is consistent with the values reported by Niu et al.^51^ (200–600 MPa) and Ramachandramoorthy et al.^52^ (about 400 MPa), both of whom investigated micropillar compression. It must be noted, however, that the former^51^ examined single crystal copper and the latter^52^ micro additive-manufactured copper pillars. These materials are expected to differ from the copper used in our study, which comes from hybrid bonding-ready patterns. Moreover, the discrepancy observed between yield stress values derived from indentation stress–strain curves and those obtained from micropillar testing is similar to previously reported comparisons.^49^

Further analysis of the force-depth curves reveals that the average envelopes from the multistep indentations resemble those from the average curves presented in Sections 3.2 and 3.3. In particular, the inflection around 7.5 μN persists, but can now be quantified in terms of stress using the indentation stress–strain formalism. As a result, we can determine that the transition observed in the force-depth curves, coinciding with the largest pop-ins, occurs at a stress of approximately 3.75 GPa.

## 4 Conclusions

The push for smaller and more efficient semiconductor devices requires length scale- and condition-relevant metrology techniques that can inform on the mechanical and thermomechanical properties of the materials involved. Herein, we have demonstrated an AFM-based protocol to gain insight into the room-temperature, nanoscale mechanical properties of Cu in hybrid bonding-ready patterns. Specifically, we have (i) quantified the statistics and characteristics of incipient plasticity and obtained nanoscale constitutive behavior for the Cu pads, and (ii) developed a multimodal AFM-based methodology for characterizing these materials at the nanoscale. The parameters obtained (Young’s modulus, yield stress, strain-hardening slope) can be effectively utilized in the design and modeling of key hybrid bonding processes, such as chemical-mechanical polishing^5^ and diffusion-bonding.^9^ Furthermore, we have outlined a roadmap for integrating an additional AFM-based material property characterization into existing AFM metrologies in the semiconductor industry. Unlike other techniques, AFM does not require extra sample preparation, allows for high-precision positioning, and can be used in- or near-line metrology applications.

Building on the findings of this study, several avenues for future research emerge. Along these lines, we emphasize the integration of CR-AFM and AFM-indentation experiments with microstructural correlative measurements such as electron backscatter diffraction and transmission electron microscopy. This integration can enhance our understanding of the relationship between microstructural states and deformation mechanisms observed in AFM experiments. Additionally, our multimodal experiments could be used to inform and compare with multiscale computational modeling approaches (e.g., molecular and discrete dislocation dynamics, crystal-plasticity finite elements) aimed at both forward and inverse material property prediction.^53^ Finally, expanding the multimodal capabilities of AFM into a high-throughput and high-speed experimental framework holds significant potential in the context of data-driven approaches, where big-data-generating experiments are increasingly demanded.^23,54^

## Supplementary Material



## Data Availability

The data underlying this study are openly available in NIST Public Data Repository at https://doi.org/10.18434/mds2-3867.^58^

## Notes

Additional information on the methodology and measurements (CR-AFM, single-step indentation, multistep indentation, AFM probe geometry) (PDF)
