# Supplementary material for: Probing the Nanoscale Onset of Plasticity in Electroplated Copper for Hybrid Bonding Structures via Multimodal Atomic Force Microscopy
Source: ACS Appl Nano Mater. 2025 Dec 9;8(50):24244–53. doi: 10.1021/acsanm.5c05142 (PMC12723640; doi:10.1021/acsanm.5c05142)
Supplement: Supplementary file 1 [file an5c05142_si_001.pdf]

Supporting Information

Probing the Nanoscale Onset of Plasticity in Electroplated Copper for Hybrid Bonding Structures via Multimodal Atomic Force Microscopy

Nicolas A. Alderete[1,2], Paresh D. Daharwal[3], Cristian V. Ciobanu[4], Gheorghe Stan[1].*

[1] Material Measurement Laboratory, National Institute of Standards and Technology, Gaithersburg, MD 20899, United States.

[2] School of Science and Engineering, The George Washington University, Washington DC 20052, United States.

[3] Advanced Packaging Technology and Manufacturing, Intel Corporation, Hillsboro, OR 97124, United States.

[4]Department of Mechanical Engineering and Materials Science Program, Colorado School of Mines, Golden, CO 80401, United States.

*Corresponding Author: gheorghe.stan@nist.gov

1. Details of the contact resonance AFM (CR-AFM) on SiO_2 and Cu pads

CR-AFM measurements were performed using photothermal excitation of the cantilever in the dual AC mode with a Vero AFM (Oxford Instruments/Asylum Research, Santa Barbara, CA, USA). A DT-NCHR probe (Nanosensors, Neuchatel, Switzerland) with a resonance frequency in air $f_1^{\mathrm{air}} = 415.8$ kHz and a spring constant $k_{\mathrm{c}} = 9.2\ \mathrm{N/m}$ was used in these measurements. CR-AFM frequency maps were generated over a $9\ \mu\mathrm{m} \times 9\ \mu\mathrm{m}$ square area encompassing a single Cu pad surrounded by SiO_2. The mapping was carried out with an applied force of $750\ \mathrm{nN}$, a frequency scan of 0.5 Hz, and a resolution of $512\ \mathrm{px} \times\ 512\ \mathrm{px}$. The fast-scan direction in CR-AFM was the same as that used in topography scanning, specifically along the cantilever's long axis. The two driving frequencies were kept apart during CR mapping at a constant difference of 120 kHz. Several CR frequency sweeps from the initial tuning states on SiO_2 and Cu regions are shown in Figure S1.

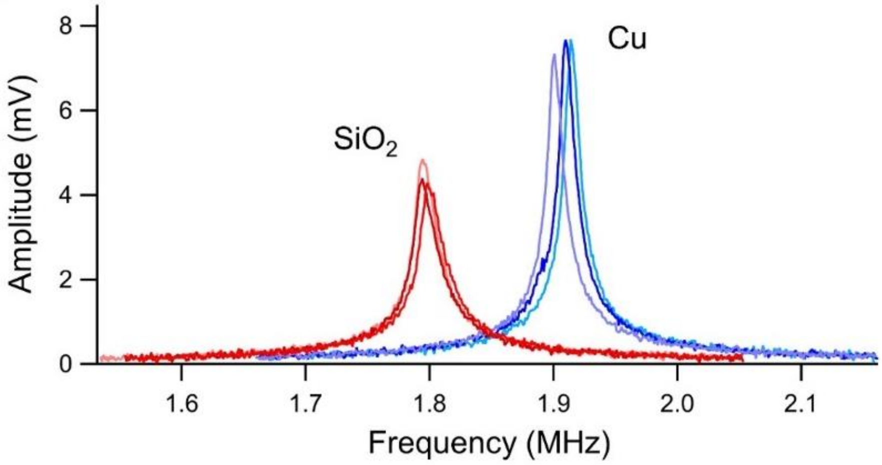

Figure S1. Measured contact resonance frequency f_{c}: sweeps over SiO_2 and Cu regions.

The measured CR frequencies were converted into indentation moduli M by following the CR-AFM analysis procedure based on a single reference material[1–3]. Briefly, we used the equation for flexural vibration along a clamped-spring coupled beam to relate the measured CR frequencies to the vertical contact stiffness, k_{vert}, of the tip-sample contact[4,5]. We modeled our cantilever as a beam with a length $L_{\mathrm{c}} = 125\ \mu\mathrm{m}$, clamped at its base, and tilted at an angle of $12°$. The tip has a length of $14\ \mu\mathrm{m}$, and is

coupled to the sample through normal k_{vert} and lateral k_{lat} springs attached at the end of the tip. We have taken $k_{\text{lat}} = 0.85 k_{\text{vert}}$ for both SiO_2 and Cu. By numerically solving these equations, we determined $k_{\text{vert}}(f_c)$ at any point of the scanned domain. We have also calculated the average value of k_{vert} over the reference SiO_2 material, $k_{\text{vert}}^{\text{ref}} = k_{\text{vert}}(f_c^{\text{SiO2,avg}})$. Finally, under the assumption of a spherical tip in contact with a flat, elastic substrate, we used Hertz contact mechanics to determine the indentation modulus M_{sample} at any point on the map relative to the indentation modulus of SiO_2, M_{ref}, as[5]:

$$M_{\text{sample}} = \frac{M_{\text{ref}}(k_{\text{vert}}/k_{\text{vert}}^{\text{ref}})^{3/2}}{1+(M_{\text{ref}}/M_{\text{tip}})(1-(k_{\text{vert}}/k_{\text{vert}}^{\text{ref}})^{3/2})}\,. \quad \text{(Eq. S1)}$$

The indentation modulus M is defined as $M = E/(1-\nu^2)$, where E and ν represent the Young's modulus and Poisson's ratio, respectively. For the reference material, SiO_2, a Young's modulus $E_{\text{SiO2}} = 72$ GPa and a Poisson's ratio $\nu_{\text{SiO2}} = 0.17$ were assumed, which correspond to an indentation modulus of $M_{\text{SiO2}} = 74.1$ GPa. For the diamond tip, the indentation modulus was taken as $M_{\text{Tip}} = 1250$ GPa, with $E_{\text{Tip}} = 1200$ GPa and $\nu_{\text{Tip}} = 0.2$. The interested reader is encouraged to consult supporting references 1-4 for varied and unique applications of CR-AFM.

2. Details of the Single-Step Indentation Analysis Methodology

Fig. S2 outlines our procedure to fit the elastic portion of the single-step indentation force-depth, P vs h, curves. First, we identified the pop-ins along the loading curve using an in-house developed algorithm, which was then visually checked to ensure accuracy and completeness. This algorithm selects the pop-ins based on the expected value ($\mu(\Delta P(h))$ and the standard deviation ($\sigma(\Delta P(h))$ of the difference between the actual curve and a loess-smoothed version of the curve ($\Delta P(h)$). As such, the algorithm is designed to search for departures that exceed the experimental noise. A sensitivity parameter k controls the threshold for these departures. Once the pop-ins are identified, we proceed with conducting

Hertz fitting of the loading curve up to the elastic limit. As described in the main text, there are two fitting parameters: E_{eff} and R. Measuring the radius of the AFM tip directly is challenging (see Supporting Information Note 7 for details), so we treated R as a fitting variable. It is important to note that different combinations of E_{eff} and R can yield the same force-depth curve, which necessitates setting upper and lower bounds for these parameters. To achieve that, we employed a two-step approach. First, we fitted the unloading segment, which is assumed to represent pure elastic unloading, between $0.9P_{\mathrm{max}}$ and $0.2P_{\mathrm{max}}$ following the well-established Oliver-Pharr (OP) procedure[6]. This fitting enables us to obtain the upper and lower bounds for the tip radius. In the OP procedure, the unloading curve is fitted as a power law of the form[6]:

$$P = A(h - h_{\mathrm{f}})^{m}, \qquad \text{(Eq. S2)}$$

where A, h_{f}, m are all fitting variables and represent a scaling factor, the final depth, and the exponent of the power law, respectively. The slope of the unloading curve (dP/dh) can be calculated from the OP fitting. The relationship between the contact area, stiffness, and effective modulus is given by[6]:

$$E_{\mathrm{eff}} = \frac{\sqrt{\pi}}{2}\frac{dP/dh}{A} \qquad \text{(Eq. S3)}$$

Here, for a spherical tip, the contact area A can be computed as:

$$A = \pi(2h_{\mathrm{c}}R - h_{\mathrm{c}}^{2}), \qquad \text{(Eq. S4)}$$

with the contact depth $h_{\mathrm{c}} = h_{\mathrm{t}} - \varepsilon[P_{\mathrm{t}}/(dP/dh)]$, and ε a geometric constant [6]. By substituting Eqs. S2 and S4 into Eq. S3, we can calculate the tip radius R by bounding E_{eff} with values from the CR-AFM measurements, $E_{\mathrm{lb(ub)}} = \tilde{E}_{\mathrm{CR-AFM}} \pm \sigma(E)_{\mathrm{CR-AFM}}$. Here, $\tilde{E}_{\mathrm{CR-AFM}}$ and $\sigma(E)_{\mathrm{CR-AFM}}$ are the mean and standard deviation of the modulus measurements from CR-AFM with the subscripts lb and ub for the upper and lower bounds, respectively. Additionally, the fitted exponent m from the Oliver-Pharr procedure was used as a check for the overall shape of the indenter, with values of 0, 1.5, and 2 for flat, paraboloid, and conical indenters, respectively. In summary, the Oliver-Pharr methodology was used only to establish bounds for the solution space during the subsequent Hertzian fitting of the loading curve

up to the first pop-in. The Hertzian fitting was conducted via least-squares minimization with variable starting points (up to the first pop-in) to determine the effective point of initial contact. This approach minimizes artifacts that may arise from surface conditions at the initial point of contact, such as roughness or oxide layers[7].

Once the values for the modulus and tip radius are obtained, the maximum shear stress at the first pop-in event can be calculated, along with the cumulative plastic excursion length δ. This is defined as the difference in displacement between the elastic response of the material and its actual elastic-plastic response:

$$\delta = h_{\mathrm{t}} - \left(\frac{3P_{\mathrm{t}}}{4E_{\mathrm{eff}}\sqrt{R}}\right)^{2/3} \quad \text{(Eq. S5)}$$

The analysis workflow is summarized in the flowchart of Fig. S2a with the relevant variables from the indentation force-depth curves highlighted in Fig. S2b.

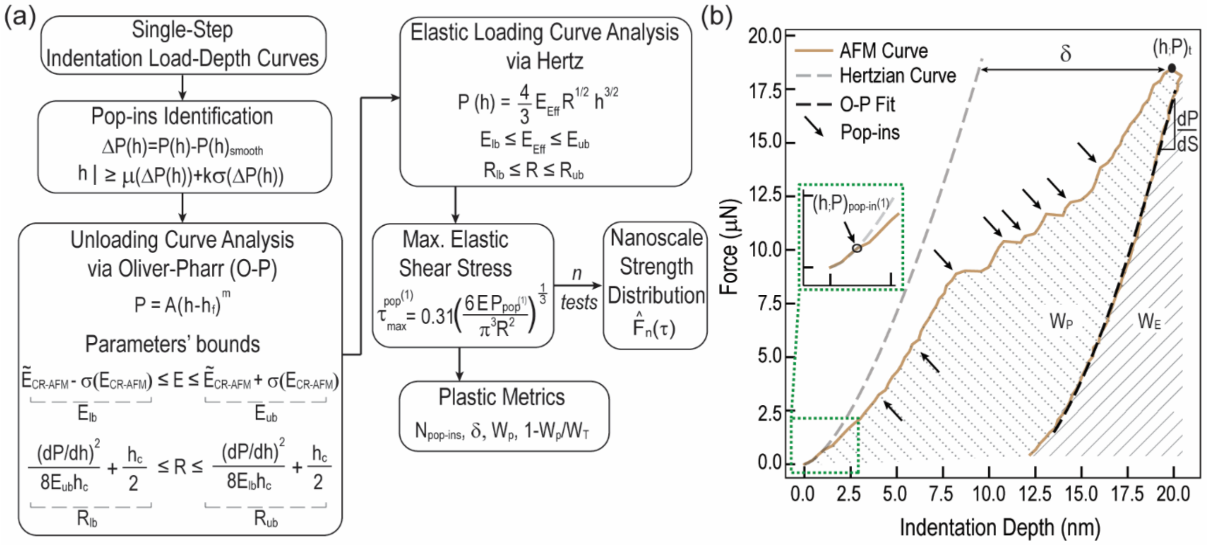

Figure S2. (a) Analysis workflow for single-step indentation curves; (b) AFM-based single-step indentation force-depth curve with relevant fits, events and variables of interest (see (a)) highlighted.

3. Details of the Multi-Step Indentation Analysis Methodology

The procedure summarized in Fig. S3 was followed to obtain indentation stress-strain curves from multi-step AFM-indentation experiments. Each unloading segment $(P-h)^{(i)}$ of a multi-step indentation curve was fitted between $0.9P_{\mathrm{t}}^{(i)}$ and $0.2P_{\mathrm{t}}^{(i)}$, with the general Hertz expression:

$$P^{(i)} = \frac{4}{3} E_{\mathrm{eff}} \sqrt{R_{\mathrm{eff}}^{(i)}}\, h^{(i)^{3/2}}, \qquad \text{(Eq. S6)}$$

where $P_{\mathrm{t}}^{(i)}$ is the maximum load for each step, and E_{eff} is the effective indentation modulus and $R_{\mathrm{eff}}^{(i)}$ the effective radius. In fitting Eq. S6, with $R_{\mathrm{eff}}^{(i)}$ as a sole fitting parameter, it was assumed that the moduli of the sample and tip remained constant (i.e., constant E_{eff}). The value of the effective radius was then used to calculate the contact radius as $r_{\mathrm{c}}^{(i)} = (3P_{\mathrm{t}}^{(i)} R_{\mathrm{eff}}^{(i)} / 4E_{\mathrm{eff}})^{1/3}$, with $(P_{\mathrm{t}}^{(i)}, h_{\mathrm{t}}^{(i)})$ being the maximum load and depth at the maximum load for each segment[8]. Pairs of stress-strain values were ultimately obtained as[9]:

$$\left(\varepsilon_{\mathrm{ISS}}^{(i)}; \sigma_{\mathrm{ISS}}^{(i)}\right) = \left(\frac{4h_{\mathrm{t}}^{(i)}}{3\pi r_{\mathrm{c}}^{(i)}} \; ; \; \frac{P_{\mathrm{t}}^{(i)}}{\pi r_{\mathrm{c}}^{(i)^2}}\right). \qquad \text{(Eq. S7)}$$

It is important to note that only the stress-strain pairs obtained from unloading fits with scores $R^2 \geq 0.7$ were retained, which means the number of stress-strain pairs may differ from the number of steps. For each experiment, a robust Theil-Sen linear regression was performed to determine the tangent stiffness, also referred to as the plastic slope E_{T}. From this, the indentation yield stress was calculated as the intersection of the plastic linear path and the 0.2 % offset elastic path. The analysis workflow is summarized in the flowchart of Fig. S3a. An example of a multi-step indentation curve is shown in Fig. S3b with fits on unloading steps and marked stress-strain pairs. The stress-strain curve obtained from this experiment is plotted in Fig. S3c along with its elastic and plastic path fits.

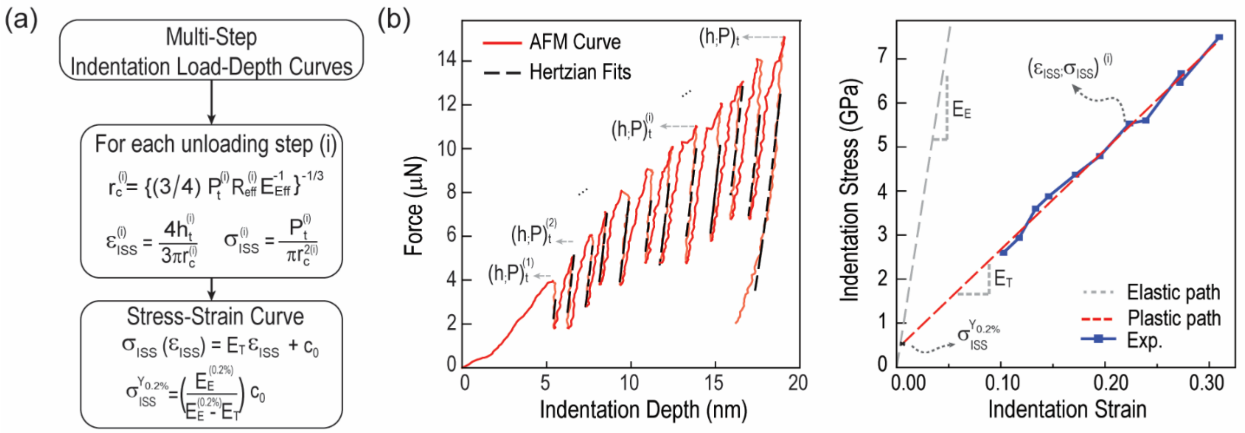

Figure S3. (a) Analysis workflow for multi-step indentation curves; (b) AFM-based multi-step indentation force-depth curve with relevant fits, events, and variables of interest highlighted; (c) the determined stress-strain curve obtained from the multi-step indentation curve shown in (b), with its elastic and plastic path fits.

4. Incipient Plasticity Characterization

Table S1 presents the experimental parameters during force-spectroscopy measurements on CuPad-1 and CuPad-2 arrays as described in Section 3.2 of the main text.

Table S1. Experimental parameters for AFM single-point indentation tests.

Array	Sensitivity (nm/V)	Cantilever Stiffness (nN/nm)	Approach/Retract Velocity (nm/s)	Number of indents	Spacing between indents (nm)
CuPad-1	510.45	527	200	64	500
CuPad-2	510.45	529	200	62	500

Figures S4 and S5 present 14 randomly selected force-indentation depth curves with parameters specified in Table S1. The detected pop-ins are highlighted with black arrows along each of these curves. The first pop-in is specifically marked with a circle, and the corresponding shear stress, indicating the

initiation of plastic deformation, is annotated. Additionally, we show the Hertzian and Oliver-Pharr fits for both the loading and unloading segments of each curve.

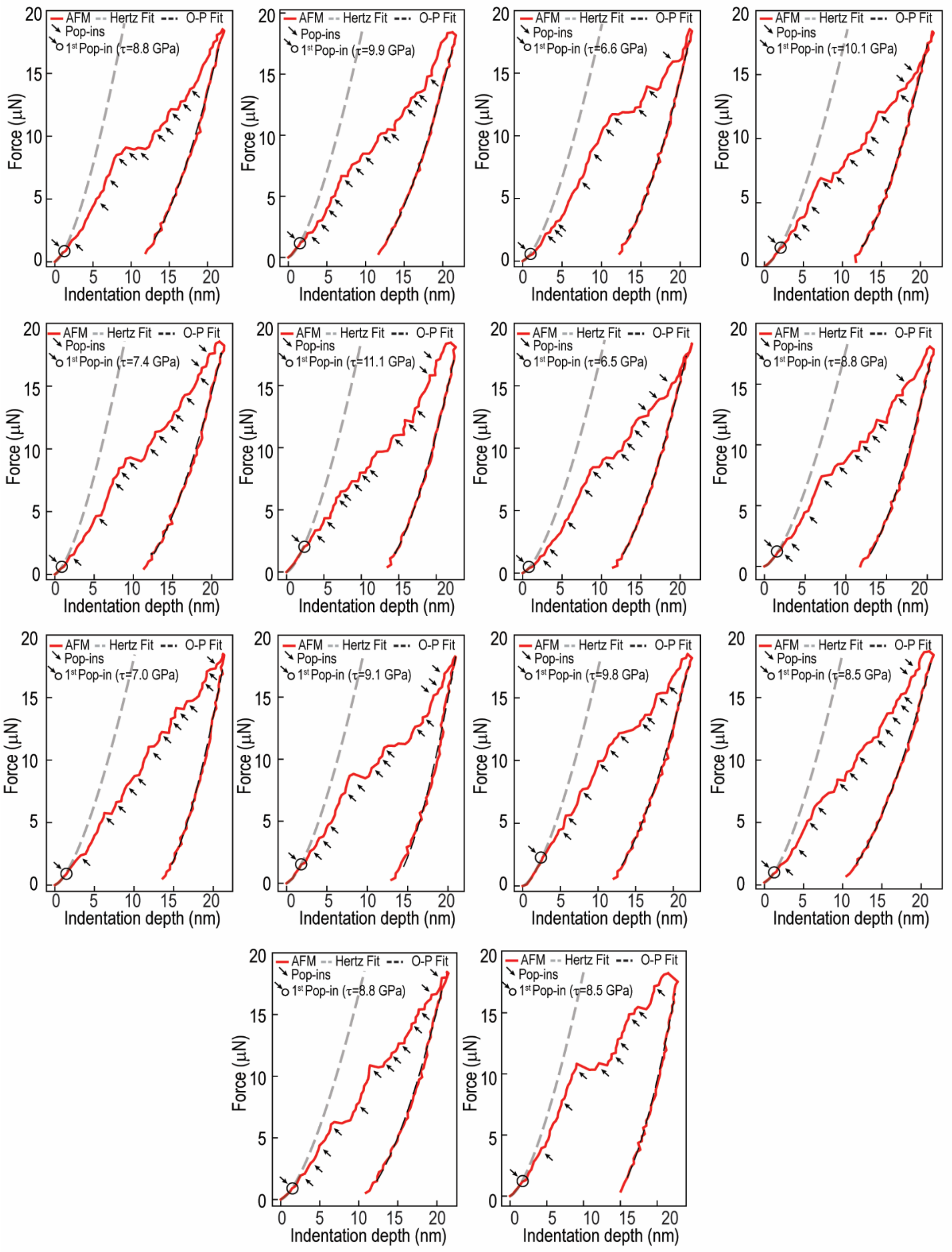

Figure S4. Force-displacement curves of single-step indentations on CuPad-1.

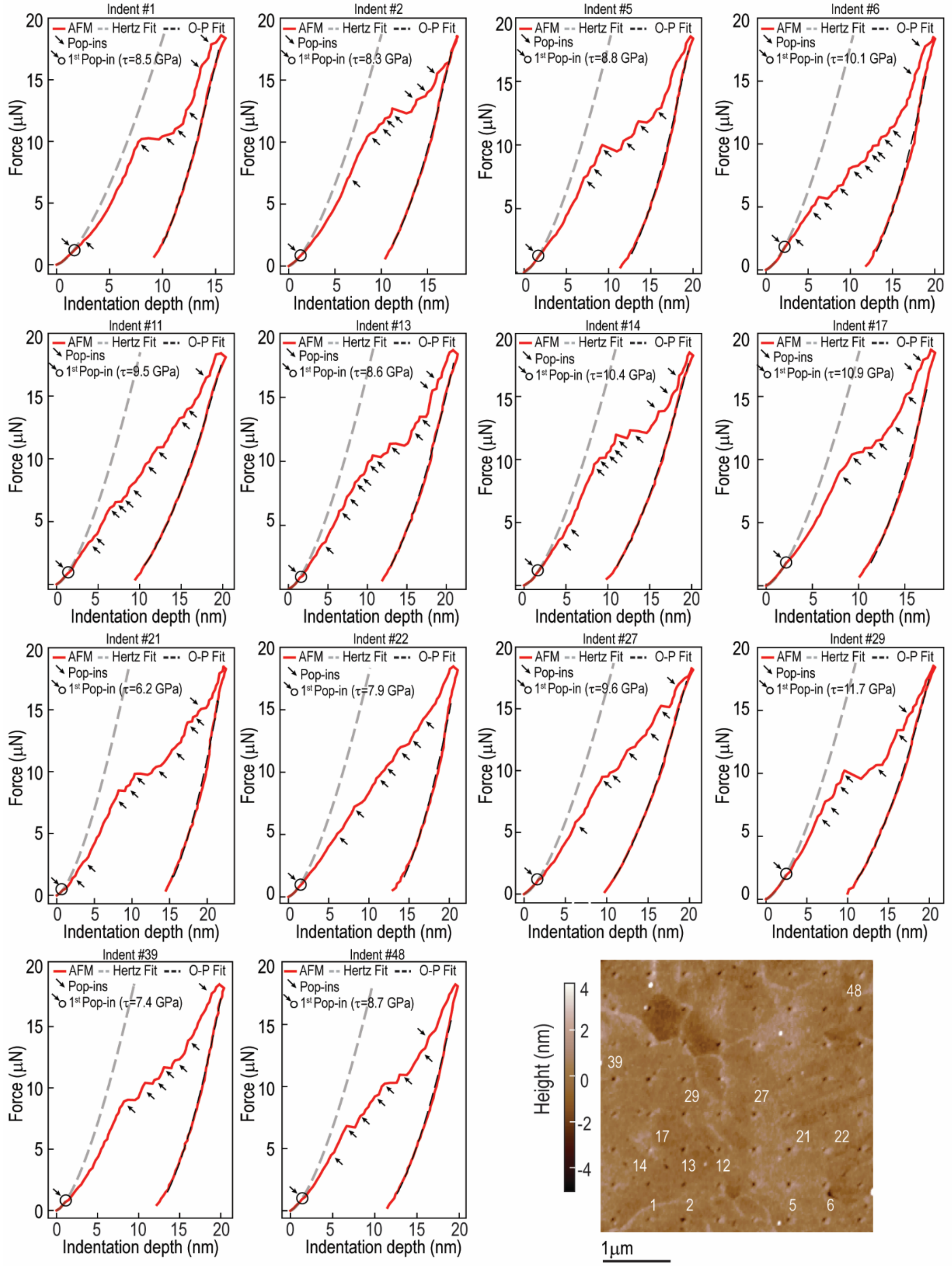

Figure S5. Force-displacement curves of single-step indentations on CuPad-2. The locations of these indents are indicated in the AFM topography image included in the figure.

5. Influence of Grain Boundaries

Table S2 presents the experimental parameters used during single-step indentation measurements to assess the effect of grain boundaries on the response of copper in hybrid bonding patterns.

Table S2. Experimental parameters for AFM single-step indentation tests within grains and on grain boundaries.

Location	Sensitivity (nm/V)	Cantilever Stiffness (nN/nm)	Approach/Retract Velocity (nm/s)	Number of indents
Grain Interior	510.45	529	200	12
Grain Boundary	510.45	529	200	17

Figure S6 and Figure S7 present a subset of randomly selected force-displacement curves from the indentation measurements performed within grains and on grain boundaries, respectively. The determined pop-ins are highlighted with black arrows along each of these curves. The first pop-in is specifically marked with a circle, and the corresponding shear stress shear, indicating the initiation of plastic deformation, is annotated. Additionally, we show the Hertzian and Oliver-Pharr fits for both the loading and unloading segments of each curve. Topographic maps after indentation are shown, with the locations of the indents corresponding to the curves highlighted.

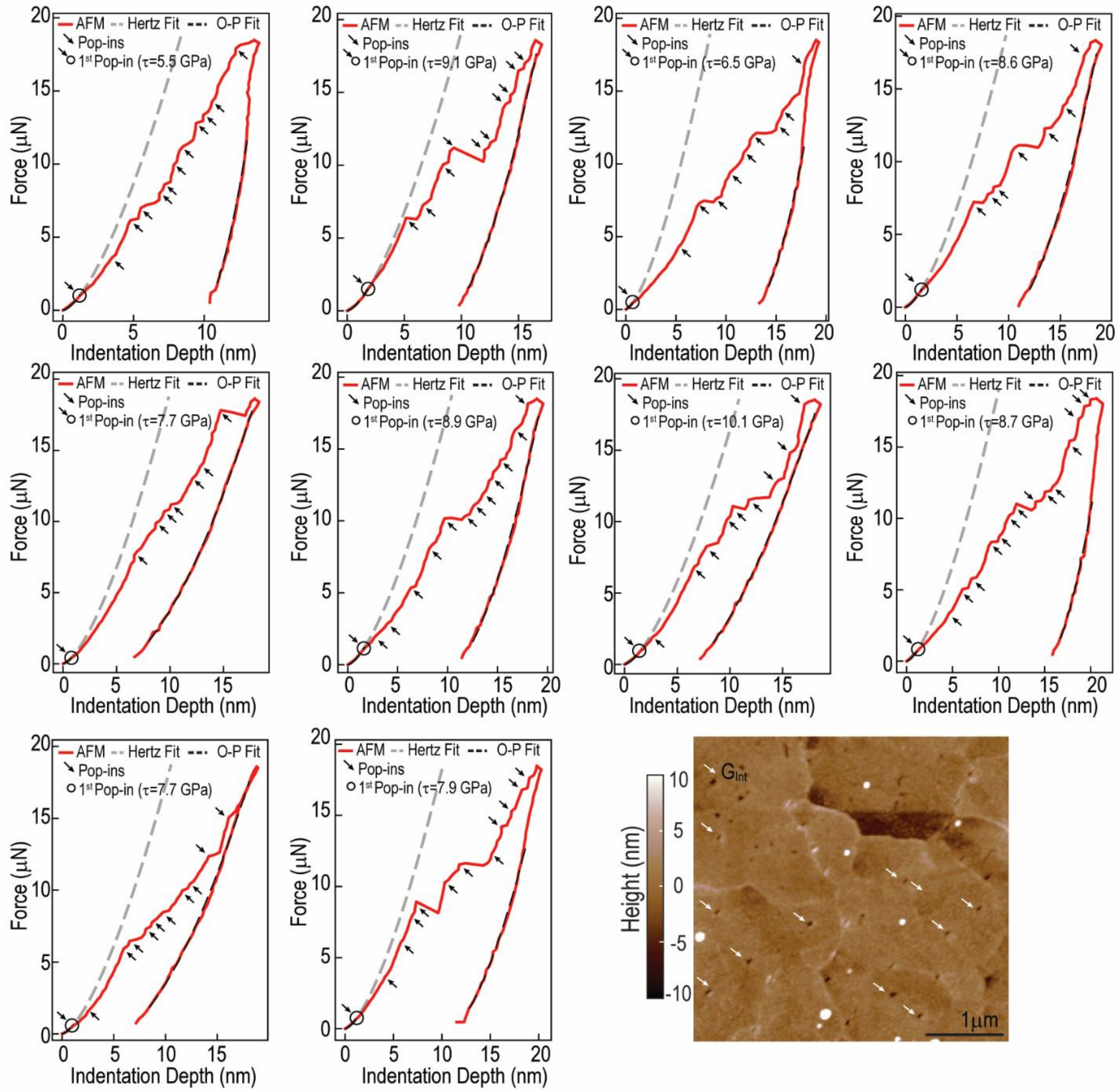

Figure S6. Force-displacement curves of indentations within grains.

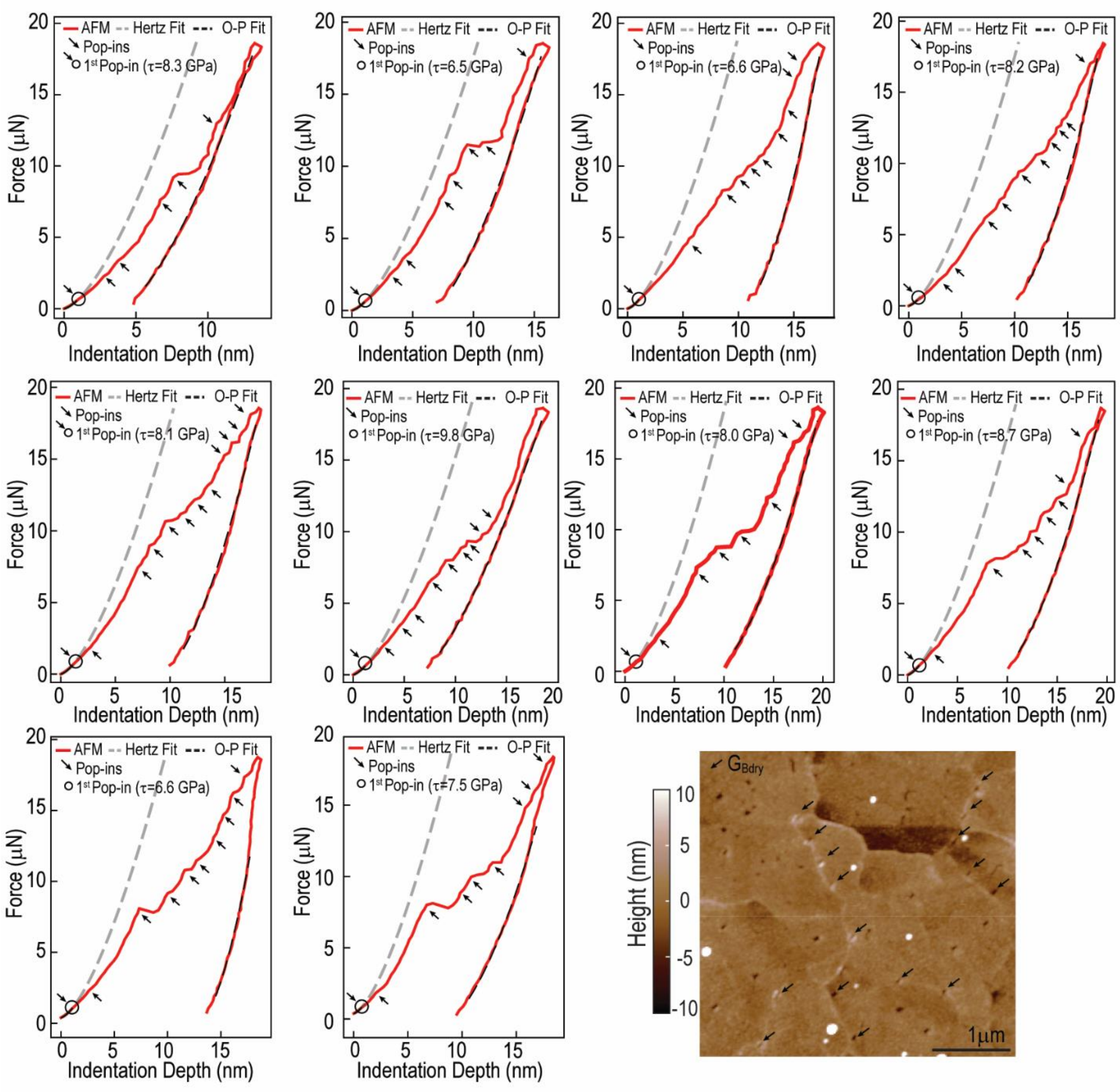

Figure S7. Force-displacement curves of indentations on grain boundaries.

6. Indentation Stress-Strain Curves

Table S3 presents the experimental parameters during multistep force-spectroscopy measurements performed to obtain the indentation stress-strain pairs presented in Section 3.4 of the main text.

Table S3. Experimental parameters for AFM multi-step indentation tests.

Inverse Optical Lever Sensitivity (nm/V)	Cantilever Stiffness (nN/nm)	Approach/Retract Velocity (nm/s)	Number of Indents
510.45	514	200	20

Figure S8 presents 6 randomly selected force-indentation depth curves, along with their derived indentation stress-strain curves, based on the parameters specified in Table S3. In addition to the AFM curves, the Hertzian fits on the unloading segments are also included. The derived stress-strain curves are shown with their elastic and plastic paths, as well as the indentation yield stress.

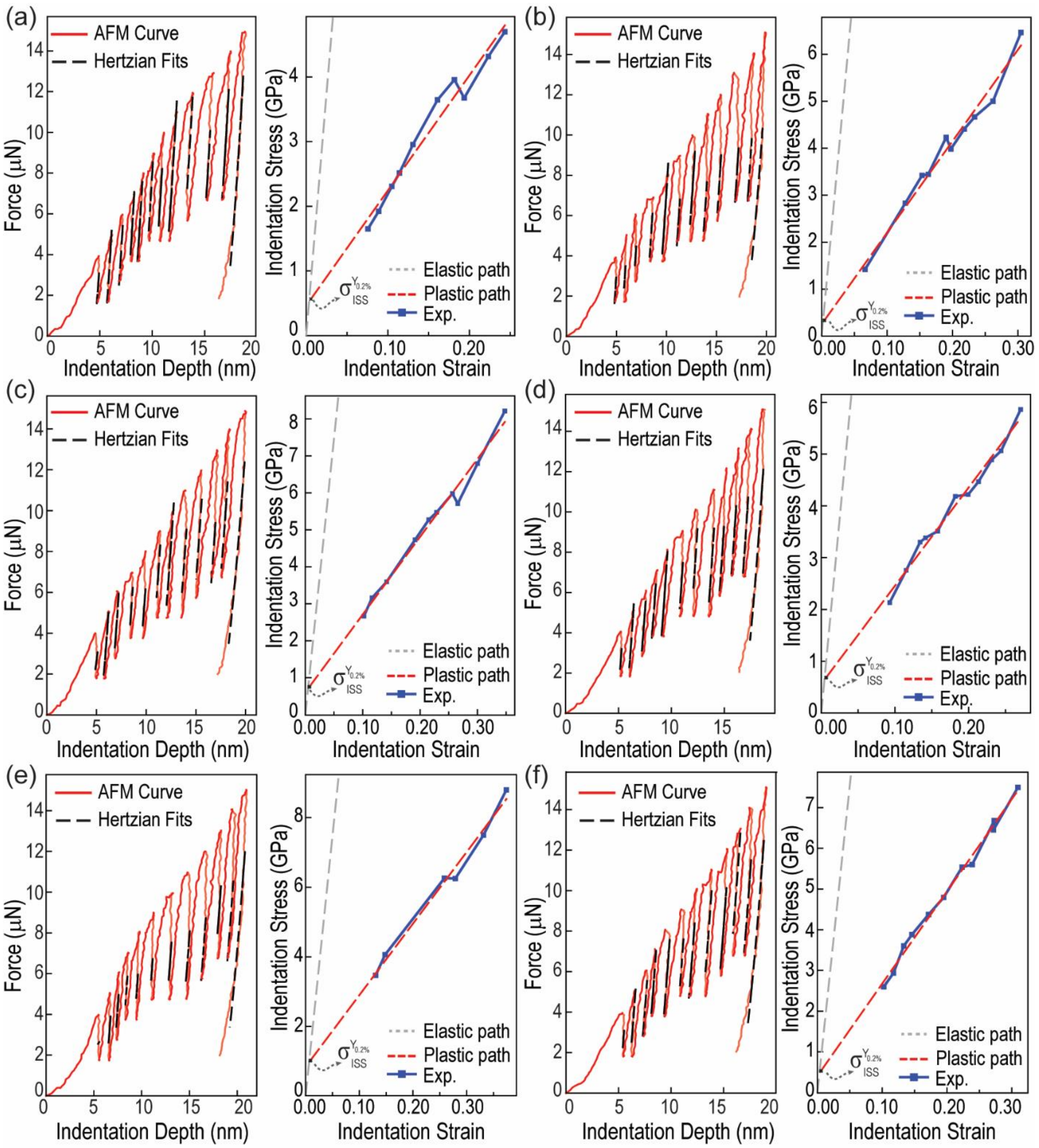

Figure S8. Force-displacement curves and derived indentation stress-strain curves from multi-step indentation measurements.

7. AFM Probe Tip-Shape Assessment

The analysis used for both single-step and multi-step indentation measurements assumes that the indenter's tip is spherical. However, ex-situ characterization of the tip before indentation is quite challenging, as handling and imaging can carry uncertainty, and more critically, they can deform and damage the tip[10]. To address this, we have opted to: i) determine the radius of the tip by fitting the elastic region of the force-indentation depth curves (as explained in Supporting Information Note 2 and Section 3.2 in the main text), and ii) characterize the shape of the tip by imaging it against a TGT1 test grating (K-TEK Nanotechnology, Wilsonville, OR, USA).

Figure S9a presents 2D and 3D AFM topographic images of the TGT1 test grating obtained before and after the single-step indentations. It can be observed that throughout our measurements, which numbered in the hundreds, the tip maintained a spherical end, with only a slight increase in tip radius observed after these numerous measurements (see also cross sections of Fig. S9b). Additionally, Figure S9c presents the statistical distribution of the tip's radius, as obtained from the Hertzian fits of the single-step indentation measurements (Sections 3.2 and 3.3 of the main text). The distribution of the radius from these fits is centered around 12.3 nm with a standard deviation of 1.4 nm. These values align with those reported by the vendor. In the case of our multi-step indentation experiments, we used a different tip than the one employed for the single-step indentations. Alongside the multi-step experiments, we also carried out a series of single-step indentations to determine the physical radius of the tip. From these measurements, we obtained a statistical distribution for the tip radius, centered at 22.0 nm with a standard deviation of 2.5 nm (see Fig. S9d). Consequently, the tip used for the multi-step indentations discussed in Section 3.4 is slightly larger than the one used for the single-step indentations reported in Sections 3.2 and 3.3 of the main text.

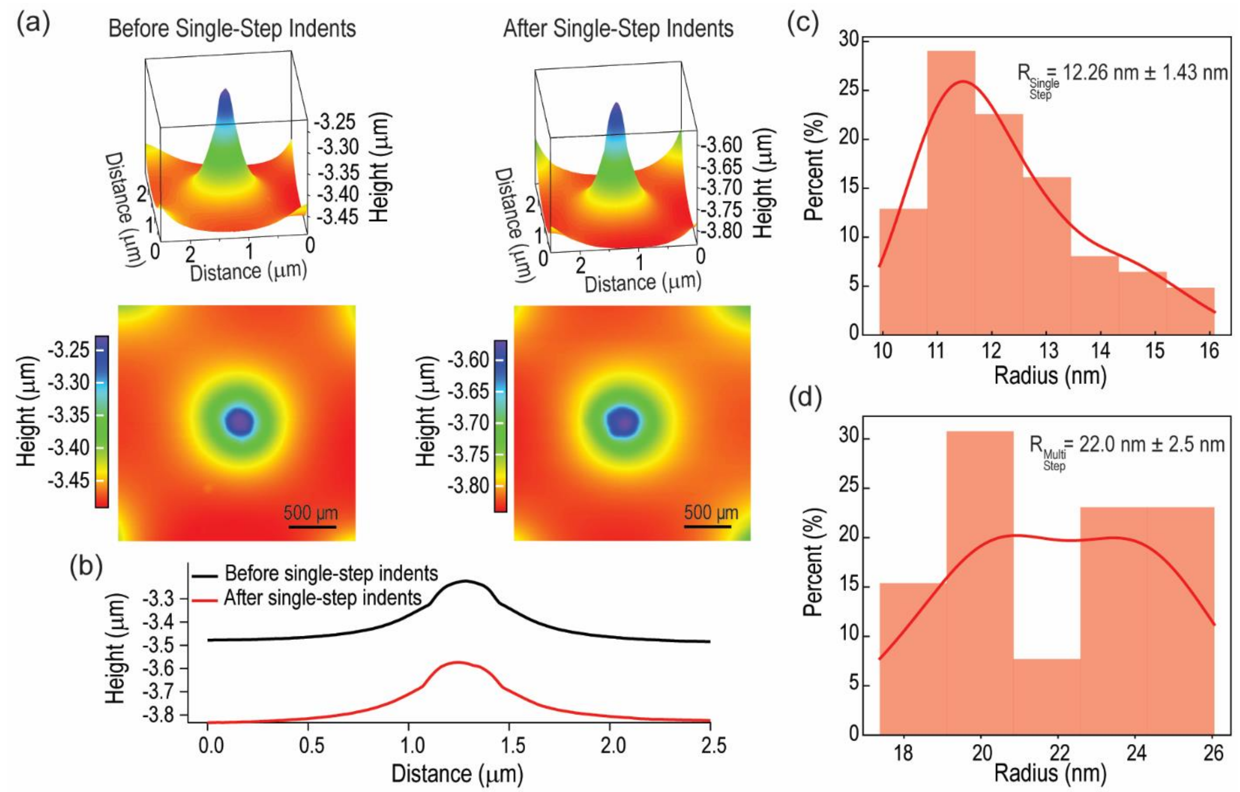

Figure S9. Shape characterization of the tip used in single-step and multi-step indentation measurements. (a) 2D and 3D topography scans of TGT1 test grating before (left) and after (right) indentations; (b) 1D height profile from a transverse cross section across the topographies shown in (a); (c) Statistical distribution of tip radius used for single-step indentations, obtained from analyzing the elastic region of the force-depth curves from single-step indentation experiments; (d) Statistical distribution of tip radius used for multi-step indentations, obtained from analyzing the elastic region of force-depth curves from several single-step indentations conducted alongside the multi-step indentation experiments.

ASSOCIATED CONTENT

The data underlying this study are openly available in NIST Public Data Repository at https://doi.org/10.18434/mds2-3867 [11].

NOTES

Certain commercial equipment, software, instruments, and materials are identified in this document. Such identification does not imply recommendation or endorsement by the National Institute of Standards and Technology, nor does it imply that the products identified are necessarily the best available for the purpose.

This work was performed with funding from the CHIPS Metrology Program, part of CHIPS for America, National Institute of Standards and Technology, U.S. Department of Commerce.

REFERENCES

(1) Stan, G.; Ciobanu, C. V.; King, S. W. Resolving the Subsurface Structure and Elastic Modulus of Layered Films via Contact Resonance Atomic Force Microscopy. *ACS Appl Mater Interfaces* **2022**, *14* (49), 55238–55248. https://doi.org/10.1021/ACSAMI.2C17962/ASSET/IMAGES/LARGE/AM2C17962_0008.JPEG.

(2) Stan, G.; King, S. W. Atomic Force Microscopy for Nanoscale Mechanical Property Characterization. *Journal of Vacuum Science & Technology B, Nanotechnology and Microelectronics: Materials, Processing, Measurement, and Phenomena* **2020**, *38* (6), 60801. https://doi.org/10.1116/6.0000544/588943.

(3) Garcia, R. Nanomechanical Mapping of Soft Materials with the Atomic Force Microscope: Methods, Theory and Applications. *Chem Soc Rev* **2020**, *49* (16), 5850–5884. https://doi.org/10.1039/D0CS00318B.

(4) Rabe, U.; Janser, K.; Arnold, W. Vibrations of Free and Surface-coupled Atomic Force Microscope Cantilevers: Theory and Experiment. *Review of Scientific Instruments* **1996**, *67* (9), 3281–3293. https://doi.org/10.1063/1.1147409.

(5) Rabe, U. Atomic Force Acoustic Microscopy. In *Applied Scanning Probe Methods II*; Bharat Bhushan, Harald Fuchs, Eds.; Springer, Berlin, Heidelberg, 2006; pp 37–90. https://doi.org/10.1007/3-540-27453-7_2.

(6) Oliver, W. C.; Pharr, G. M. An Improved Technique for Determining Hardness and Elastic Modulus Using Load and Displacement Sensing Indentation Experiments. *J Mater Res* **1992**, No. 6.

(7) Pathak, S.; Shaffer, J.; Kalidindi, S. R. Determination of an Effective Zero-Point and Extraction of Indentation Stress–Strain Curves without the Continuous Stiffness Measurement Signal. *Scr Mater* **2009**, *60* (6), 439–442. https://doi.org/10.1016/J.SCRIPTAMAT.2008.11.028.

(8) Donohue, B. R.; Ambrus, A.; Kalidindi, S. R. Critical Evaluation of the Indentation Data Analyses Methods for the Extraction of Isotropic Uniaxial Mechanical Properties Using Finite Element Models. *Acta Mater* **2012**, *60* (9), 3943–3952. https://doi.org/10.1016/J.ACTAMAT.2012.03.034.

(9) Pathak, S.; Kalidindi, S. R. Spherical Nanoindentation Stress–Strain Curves. *Materials Science and Engineering: R: Reports* **2015**, *91*, 1–36. https://doi.org/10.1016/J.MSER.2015.02.001.

(10) Flater, E. E.; Zacharakis-Jutz, G. E.; Dumba, B. G.; White, I. A.; Clifford, C. A. Towards Easy and Reliable AFM Tip Shape Determination Using Blind Tip Reconstruction. *Ultramicroscopy* **2014**, *146*, 130–143. https://doi.org/10.1016/J.ULTRAMIC.2013.06.022.

(11) Stan, G.; Alderete, N. Elastic-plastic AFM indentation on copper, **2025**, National Institute of Standards and Technology. https://doi.org/10.18434/mds2-3867
